# Remote Follow-Up Using Smartphone Apps, Telemedicine, and Wearable Activity Sensors After Joint Replacement: A Narrative Review

**DOI:** 10.7759/cureus.96693

**Published:** 2025-11-12

**Authors:** Ali Soffar, Ahmed Elkohail, Abdelrahman Sayed, Mohamed Elbanna, Kerementi Othieno-P'Otonya, Mohammad Monir, Hassan A Kassem, Ahmed E Abdellatif

**Affiliations:** 1 Trauma and Orthopaedics, Princess Royal University Hospital and Orpington Hospital, King’s College Hospital NHS Foundation Trust, London, GBR; 2 Trauma and Orthopaedics, Princess Royal University Hospital, King’s College Hospital NHS Foundation Trust, London, GBR; 3 Trauma and Orthopaedics, Cardiff University Hospital, Cardiff, GBR; 4 Trauma and Orthopaedics, Epsom and St Helier University Hospitals NHS Trust, London, GBR; 5 Trauma and Orthopaedics, King’s College Hospital NHS Foundation Trust, London, GBR; 6 Trauma and Orthopaedics, General Organization for Teaching Hospitals and Institutes, Cairo, EGY; 7 Critical Care Medicine, King’s College London, London, GBR; 8 Trauma and Orthopedics, Andalusia Hospital, Jeddah, SAU

**Keywords:** joint replacement, remote follow-up, smartphone, telemedicine, wearable activity sensors

## Abstract

Digital health tools such as smartphone applications, telemedicine platforms, wearable activity sensors, and emerging smart implants are positively influencing postoperative follow-up after joint replacement. This narrative review synthesizes recent advances and evidence across these modalities. Smartphone apps consistently improve patient engagement, adherence, pain management, and functional recovery, while enabling scalable data capture. Wearables provide objective, continuous measures of mobility and range of motion and have shown acceptable accuracy in arthroplasty populations. Combined app-plus-sensor programs, including self-directed rehabilitation, achieve clinical outcomes comparable to standard care in randomized trials and can reduce costs and in-person visits. Telemedicine and home-based telerehabilitation demonstrate noninferiority to face-to-face physiotherapy for total joint arthroplasty, with high patient satisfaction and logistical advantages. Smart implants, though earlier in clinical adoption, yield unique biomechanical insights that may personalize rehabilitation and enable early detection of complications. Key challenges include heterogeneous study designs and metrics, device placement-dependent accuracy, digital literacy and connectivity barriers (especially among older adults or rural patients), data security and regulatory considerations, and technical constraints for implant power and wireless communication. Overall, remote follow-up using apps and wearables is a credible adjunct, or alternative, to conventional pathways after joint replacement; scaling impact will require standardized outcomes, interoperability, robust privacy safeguards, and prospective trials that link digital signals to meaningful clinical endpoints.

## Introduction and background

Technological changes with artificial intelligence (AI) and sensor technology are affecting orthopedic surgery care [1]. Sensor technology is becoming more common in orthopedics; its practical application has not progressed as quickly as in other medical fields [2,3]. Digital health interventions have been shown to identify postoperative complications sooner, improve recovery, and offer a safe and patient-acceptable follow-up [4-6].

Artificial and advanced devices can help with delivery for more precise and tailored care [7]. New developments in technologies that use computer-guided, robotic-assisted, and navigational input have led to the widespread usage of AI devices in orthopedic surgery [8]. The use of telemedicine for patient care was greatly expedited by the COVID-19 pandemic [9,10].

Wearable devices are increasingly used in orthopedic patient care as more patients adopt them [11]. Compared to traditional postoperative care, remote patient monitoring after surgery, utilizing technologies such as sensors and wearable devices, can reduce health-related costs and improve patient outcomes [12].

These tools can help in remote assessment with objective data analysis [13]. Another digital advancement is the smart implant, which is a digital device that enables more precision and tailored care [14]. However, there are frequently obstacles to access, such as issues with accessibility, privacy, security, and the target population’s lack of acceptance and perception of the benefit of digital health technologies [15-17]. In this narrative review, we aim to highlight the digital innovations in follow-up after joint replacement surgery, highlighting the technological changes with AI and sensor technology affecting orthopedic surgery care.

## Review

Methodology

We conducted a narrative synthesis of contemporary approaches to remote follow-up after total joint arthroplasty (total knee arthroplasty (TKA)/total hip arthroplasty (THA)), spanning smartphone/mobile apps, wearable activity sensors, telemedicine/telerehabilitation, and smart implants/instrumented prostheses. We searched PubMed/MEDLINE and Scopus for English-language studies published between January 2015 and June 2024, supplemented by citation chasing of key reviews. Core search concepts combined terms for the population (arthroplasty, hip, knee), technology (smartphone, mobile app, telemedicine, video visit, telerehab, wearable, accelerometer, activity tracker, smart implant, instrumented prosthesis), and outcomes (follow-up, rehabilitation, pain, function, range of motion, gait, adherence, satisfaction, cost, accuracy, validation).

We included randomized trials, prospective/retrospective cohorts, and validation studies of apps/wearables/telemedicine/smart implants used postoperatively in adult TKA/THA. We excluded case reports, pediatric or non-arthroplasty cohorts, and studies without a remote monitoring or follow-up component.

Extracted endpoints were organized into the following harmonized domains: (1) engagement/adherence and satisfaction; (2) pain, opioid use, complications detection; (3) function/range of motion (ROM) and gait/mobility; (4) device accuracy/validity (e.g., step count, ROM, telemetric load); and (5) utilization/costs (e.g., visits avoided, resource use). Smart implant reports were additionally summarized for in vivo load/kinematics and rehabilitation-relevant biomechanics.

Given heterogeneous interventions, devices, and metrics, a meta-analysis was not attempted. Instead, we performed a qualitative, narrative synthesis, grouping results by technology category and emphasizing randomized evidence, consistent signals across cohorts, and clinically meaningful effect directions. Where available, we highlighted device placement effects and measurement error bounds for validation studies. We appraised each study’s design, sample size, follow-up, comparator, measurement methods, and reporting of funding/conflicts to contextualize bias risk. For accuracy studies, we noted reference standards and limits of agreement. Disagreements in study selection or interpretation were resolved by consensus. This review did not require ethics approval.

Smart implants

Smart implants are advanced implantable devices that offer both therapeutic advantages and diagnostic functions. These innovative implants have the potential to personalize medical treatments, enhance patient care, improve results, and simultaneously lower healthcare expenses [14].

Smart implants offer unique insights into the body’s internal environment, providing objective, quantitative data unavailable through other means. This information enables personalized treatments, facilitates care transitions, and allows for earlier detection of adverse events [18,19].

Smart implants can be used to evaluate the healing of fractures and identify several issues, including periprosthetic and other musculoskeletal infections, aseptic loosening in total joint arthroplasty, and more [20-22]. Crucially, determining the stage of fracture healing allows for the development of an appropriate postoperative plan for patients, including ROM allowances and weight-bearing restrictions. This assessment also facilitates early detection of non-unions [23].

Fracture healing values are transmitted to a patient’s smartphone via an implanted data logger on the plate. By detecting mechano-acoustic waves and sending them to an external coil, smart implants have shown in experimental settings that they can detect implant loosening and osteointegration in THA [24].

Knee Applications

Regretfully, 15-30% of TKA patients are thought to have ongoing pain, disability, and functional restrictions after surgery [25,26]. To evaluate a patient’s recovery, physical function must be evaluated by in-office functional testing and physical examinations, as well as patient-reported outcomes (PROMs). However, as this necessitates patients visiting the office, compliance rates might be as low as 35% after a year [27-29].

TKA is commonly performed and typically carries a low complication rate. Postoperative knee biomechanics, which drive ROM, implant longevity, and long-term outcomes, depend heavily on surgical technique and prosthesis design. The value of the data collected from these devices, such as pressure, angle, motion, and stresses at the implant, can inform better implant design and selection as well as refine the surgical approach, thereby improving patient results. Accordingly, smart knee implants are pivotal tools for elucidating knee biomechanics [30].

In a prospective cohort of 130 TKA recipients implanted with a smart knee (Persona IQ®, with embedded accelerometer/gyroscope), Yocum et al. examined correlations between continuously captured gait/kinematic metrics (e.g., step count, walking speed, functional knee ROM) and clinic-measured ROM and PROMs. Correlations between implant-derived kinematics and PROMs were weak, indicating that passive implant telemetry provides objective information distinct from questionnaires. The authors noted that daily, real-world measurements may help identify poor recovery earlier and complement routine visits [31].

Kutzner et al. used fully implanted telemetric knee prostheses in nine TKA patients 15 ± 7 months postoperatively to measure in vivo tibiofemoral contact forces during ergometer cycling at varied power (25-120 W), cadences (40/60 rpm), and seat heights, and compared them with walking. Cycling forces were lower than walking, increased linearly with power, were smaller at higher cadence, and lower seat height raised posterior shear. These implant-borne measurements provided objective loading profiles that can inform rehabilitation dosing and activity counseling after TKA [32].

After TKA, data from smart knee implants map the loads the joint experiences across activities. During level walking, peak loads typically reach about 1.8-2.6× body weight and are centered over the tibial tray. Treadmill walking tends to lower these loads versus hard-floor walking, but faster speeds raise them. Descending stairs produces the greatest forces of up to ~4.0× body weight. Standing up from a chair also yields substantial peaks of ~1.5-2.5× body weight. By comparison, stationary cycling imposes relatively light loads (~1.0× body weight), whereas a golf swing can approach ~4.0× body weight [33-35].

The integration of sensors, signal conditioning electronics, and telemetry within the patellar implant is complicated by the small size of the patellofemoral joint. Historically, the development of smart patellar implants utilizing conventional technology, such as strain gauges, has been impeded by this space constraint. However, recently, a smart patellar implant that can measure patellofemoral forces was created [36].

Hip Applications

In 1966, the first innovative THA was performed. A unique three-part femoral component with strain gauges in the neck had to be designed and fabricated. Lead wires ran percutaneously from the implant to an external data logger, and the prosthesis was connected. It took 10 years for the next-generation wireless prosthesis to be developed and used in clinical settings [37,38]. As a research tool, the data gathered from these implants have provided valuable insights to address various clinically relevant questions [36,39].

Damm et al. used an instrumented THA with telemetric transmission to measure in vivo contact forces and friction moments during level walking in eight subjects about three months postoperatively. They computed the three-dimensional coefficient of friction across the entire gait cycle, reporting peak contact forces ~248% BW and friction moments ~0.26% BW·m, and discussed how friction-induced moments can endanger cup fixation, information that is relevant to rehabilitation dosing and device testing/design. The study demonstrated that implant-borne sensing resolves gait cycle-specific mechanics directly in patients [40].

**Figure 1:**
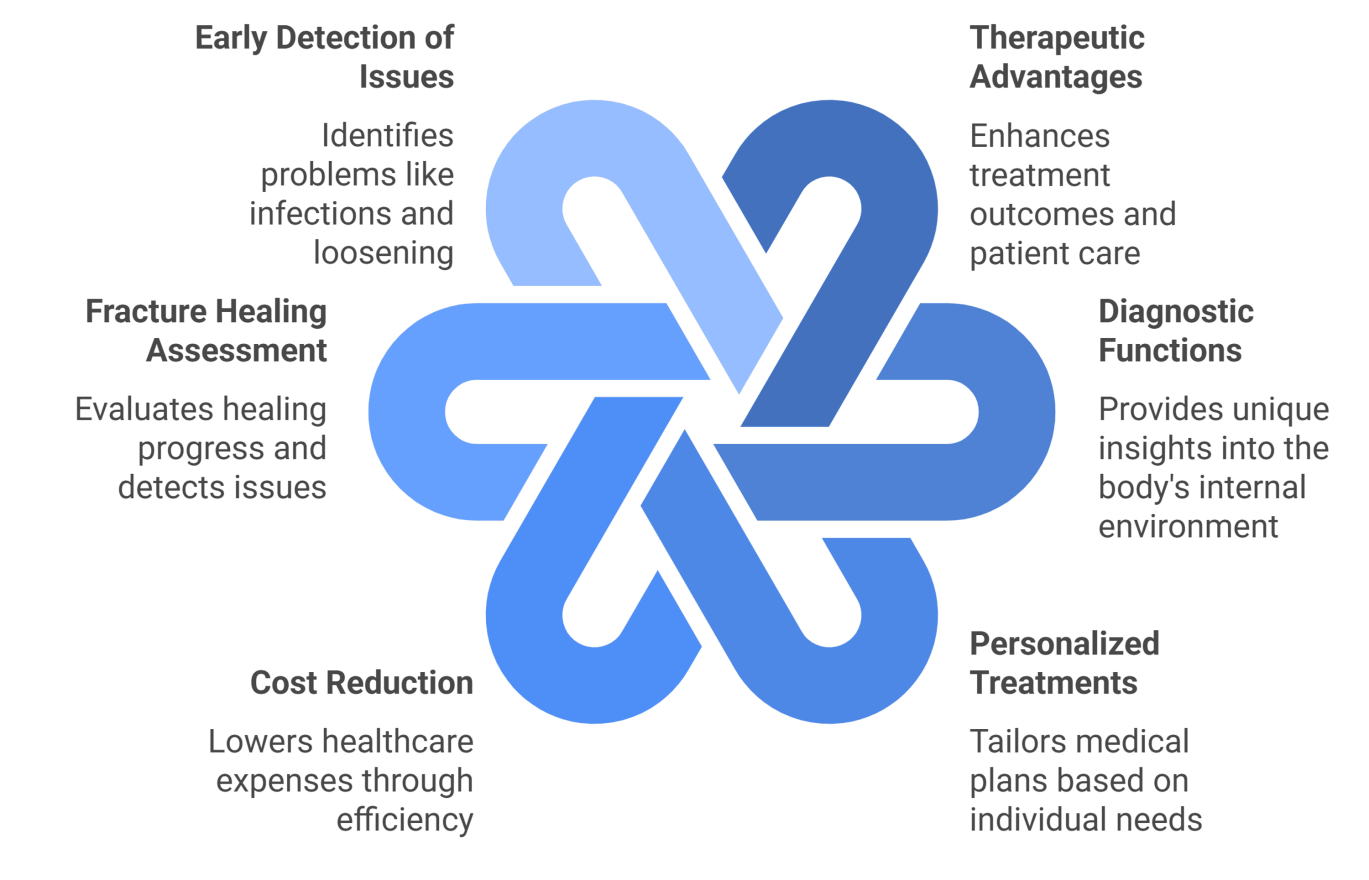
The multifaceted benefits of smart implants. The figure created by the authors summarizes the benefits of smart implants in orthopedic practice. Sources: [14-36].

Bergmann et al. published standardized in vivo load sets for THA derived from instrumented implants that synchronously measure contact forces and friction moments. Using data from 10 patients across demanding daily activities, they provided reference load time courses and cycle counts to enable more realistic preclinical testing and comparative benchmarking of implants/bearing couples. Clinically, such standardized loads can inform activity recommendations and help frame risk assessment for fixation, wear, and loosening (Figure 1) [41].

Wearable sensors and mobile outpatient monitoring

Sensors are finding a variety of uses in patient care as more people incorporate wearable technology into their everyday routines. These devices in orthopedic surgery include fitness trackers, smartwatches, and motion and pressure sensors [11].

The combination of advanced sensors with AI, big data analytics, and machine learning has enabled the development of SMART (Self-Monitoring Analysis and Reporting Technology) orthopedic devices [42,43].

Wearable sensors can capture clinically meaningful postoperative rehabilitation data, including after humeral head/proximal humerus fractures. In a recent multicenter prospective study, 13 elderly patients underwent open reduction and internal fixation for complex proximal humerus fractures and wore paired accelerometer-based trackers. Mean shoulder elevation angles remained low (11-23°), while daily elevation events varied widely (547-5,756) and increased roughly threefold with immediate mobilization, indicating feasible, protocol-sensitive monitoring that supports data-informed postoperative care [44].

Mobile Monitoring

Research has explored the efficacy of mobile applications in aiding patient rehabilitation post-arthroplasty, demonstrating beneficial outcomes such as reduced pain, improved ROM, enhanced physical function, and a better health-related quality of life [45]. Mobile apps are utilized in orthopedic surgery to engage patients across various procedures [45-48]. Given that hip and knee total joint arthroplasty volumes are forecast to increase 174% and 673% by 2030, mobile applications are particularly compelling in this area [49].

Mobile apps, when deployed in total joint arthroplasty care, have shown promise in enhancing patient satisfaction, improving adherence to perioperative protocols, and increasing engagement across the treatment pathway [45]. For instance, the PainCoach app demonstrated improved management and reduced opioid consumption within two weeks post-TKA [50]. The Moves app achieved a satisfactory follow-up data collection rate of 68% from patients [51].

Research has also examined how accurately smartphones and wearable devices measure physical activity. For example, the Dr. Goniometer app has demonstrated strong reliability and validity for remotely tracking knee ROM after TKA [52]. Another study observed that an Apple iPhone 6 and a Fitbit Charge HR exhibited an acceptable error rate (below 30%) in step count measurements. This was found when the iPhone 6 was worn on the contralateral hip and the Fitbit Charge HR on the contralateral ankle, both two weeks after surgery [53].

Two randomized controlled trials reported that self-directed rehabilitation delivered via a wearable device paired with a smartphone app was not inferior to standard postoperative care after TKA [54,55]. Together, these results indicate that such digital combinations can effectively replace or complement traditional rehabilitation in both inpatient and home settings [56].

A review of 31 studies (July 2015 to June 2024) on smartphone apps and wearables for post-TKA monitoring found benefits across all categories. App-based studies (17/18) reported higher satisfaction, adherence, improved gait, pain control, functional gains, and cost savings. Wearable-only studies (7/8) showed accurate monitoring, effective gait/motion analysis, better recovery, earlier function return, cost reduction, and guided pain management. Combined approaches (all 5) reinforced these benefits, particularly satisfaction and mobility at three months. Randomized trials confirmed their accuracy, clinical utility, and cost-saving potential [45].

**Figure 2:**
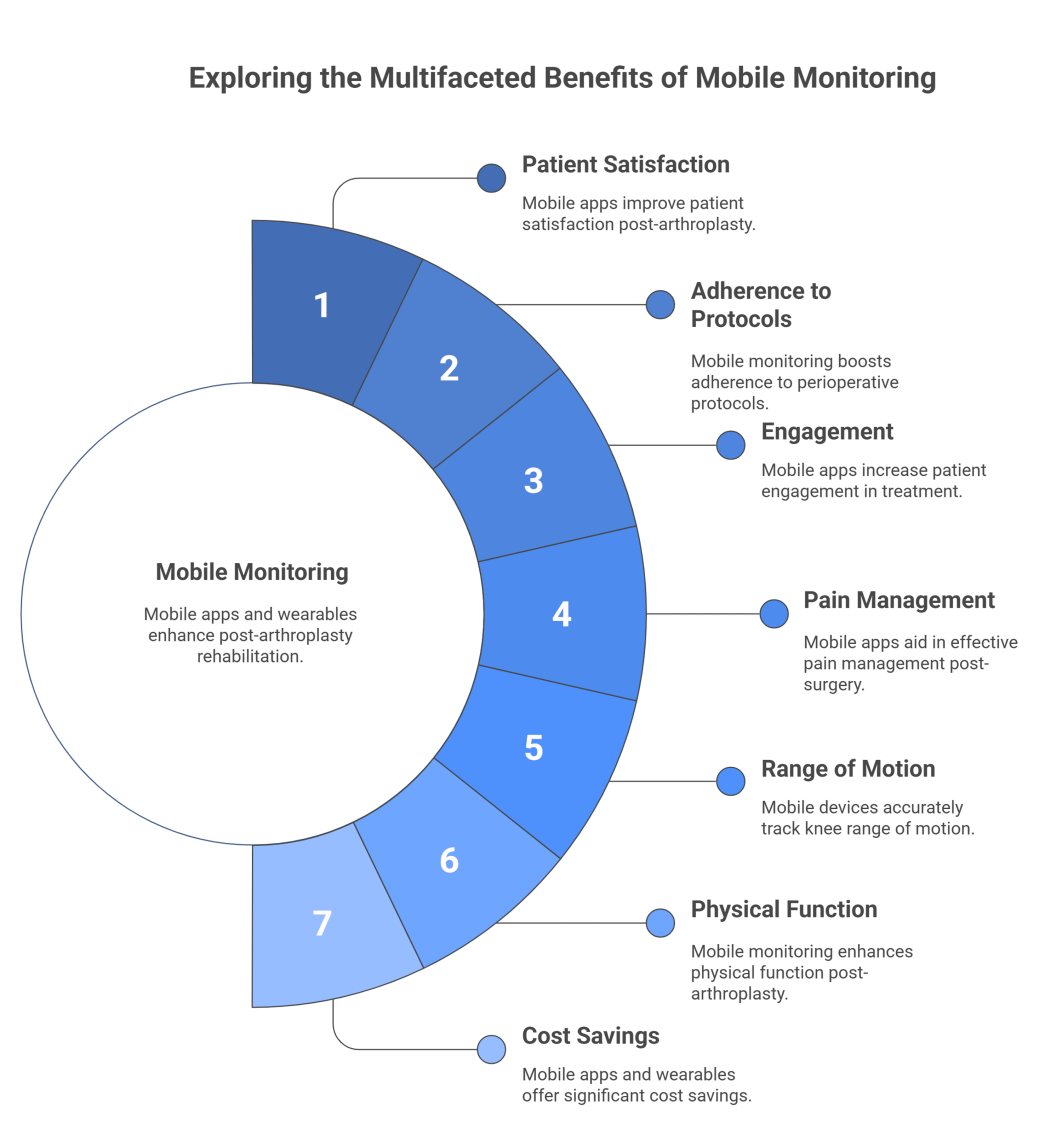
Exploring the benefits of mobile monitoring. The figure was created by the authors exploring the benefits of mobile monitoring. Sources: [45-57].

Another study evaluated smartphone apps and wearables for monitoring recovery after TKA. From 2,119 records, 58 studies qualified: 25 app-only, 25 wearable-only, and eight combined. Across outcomes, i.e., satisfaction, adherence, function, pain, and gait, most app studies showed higher satisfaction, better pain control via medication scheduling, functional gains, and cost savings. Wearables provided accurate monitoring and effective gait analysis, supporting return to function and lower costs. Combined approaches reinforced these benefits, emphasizing adherence and mobility by three months (Figure 2) [57].

Telemedicine and telehealth

Telemedicine involves delivering medical services through telecommunication technologies. Telehealth, however, encompasses additional services beyond the direct physician-patient interaction [7]. Remote assessment of orthopedic patients, particularly those with chronic conditions, is possible through online video consultations (OVCs) [58].

OVCs deliver patient satisfaction and patient-reported outcomes comparable to in-person visits, while lowering costs such as travel. They work especially well for initial outpatient evaluations of non-urgent conditions, covering history taking, digital range of motion, and functional assessments, and are also effective for postoperative follow-ups, including wound and ROM checks, rehabilitation visits, and private patient-physician discussions [59-61].

Multiple studies have shown that home-based telerehabilitation after primary total joint arthroplasty delivers outcomes comparable to traditional, in-person physiotherapy. Across multiple studies, remote programs have matched standard care on key measures, such as functional recovery and pain, while offering added advantages in convenience, access, and continuity of care. Collectively, these findings support telerehabilitation as a viable, noninferior alternative to face-to-face physical therapy for patients recovering at home [62-65].

Patient satisfaction is a key factor in assessing the effectiveness of telerehabilitation, and concerns have been raised about the challenges of technology adoption, especially among older adults. Nevertheless, research indicates that patients generally have positive experiences and satisfaction with telerehabilitation, and a lack of technological proficiency does not significantly detract from their overall satisfaction [66,67].

Limitations

Many patients may not own compatible devices or feel confident using the technology required for video visits. In addition, both patients and clinicians depend on a stable, reliable internet connection, which is often lacking in rural or underserved areas [68].

Safeguarding patient privacy and complying with health data laws are essential, demanding secure platforms and rigorous practices. Telemedicine also encounters regulatory barriers, including gaps around required hardware and software standards, protections for patients and data, insurance coverage, and reimbursement policies [69].

Several key limitations prevent the widespread clinical use of smart implants. Significant modifications are required to integrate sensors into existing implants, and considerable technical challenges remain with power, energy storage, and wireless communication. Furthermore, the use of percutaneous wires presents a risk of infection and limits patient mobility. Battery-powered devices are hindered by their large size and limited lifespan, while the overall complexity of these systems contributes to low reliability and frequent failures [36].

## Conclusions

Remote follow-up utilizing smartphone applications and wearable activity sensors presents a promising adjunct or, in some cases, an alternative to conventional postoperative monitoring and rehabilitation protocols following joint replacement surgery. Evidence indicates that these technologies are capable of producing clinical outcomes that are comparable to, and sometimes even better than, standard care. This shift toward digital monitoring offers potential cost savings and is associated with high patient satisfaction. The distinct technologies play complementary roles in patient recovery: smartphone apps are effective for providing pain management guidance, exercise instructions, and direct communication channels. In parallel, wearable sensors deliver objective, quantifiable data on a patient’s movement and physical activity levels. This combination of guided recovery and objective measurement helps support patients through their rehabilitation process. To fully realize the benefits of digital postoperative care, essential challenges must be addressed, including study heterogeneity, ensuring device accuracy, promoting patient adherence, and safeguarding privacy. Fully integrating these tools into routine orthopedic practice will require continued innovation. Furthermore, rigorous research is necessary to refine and tailor these digital tools to meet individual patient needs, ensuring their effectiveness and widespread adoption.
